# Completely Displaced Femoral Neck Stress Fracture in a Young Male Soldier With Almost No Functional Impact: A Case Report

**DOI:** 10.7759/cureus.33629

**Published:** 2023-01-11

**Authors:** Abdulaziz Alhawas, Munira Abahussain, Shahad G Alghamdi, Abdullah Alfarhan

**Affiliations:** 1 Orthopedic Surgery, King Fahad University Hospital, Alkhobar, SAU; 2 Orthopedic Surgery, King Fahad Specialist Hospital, Dammam, SAU; 3 Medicine, Imam Abdulrahman Bin Faisal University, Dammam, SAU; 4 Orthopedics, King Fahad Military Medical Complex, Dammam, SAU

**Keywords:** stress fractures, military stress fracture, completely displaced stress fracture, neck of femur, proximal femur

## Abstract

Stress fracture in general is a well-known condition that can be found commonly in healthy athletes and military personnel. These fractures occur due to bone fatigue in response to the repetitive stress of exercise.

Although few reports on stress fractures among military personnel and active young athletes can be found in previous literature, we present this case report of a young military soldier that had a completely displaced neck of femur fracture with no functional limitation for seven days prior to seeking medical advice; the patient was operated on and followed for three years after and did not develop complications associated with this kind of fracture. A reduction of neck of femur fracture and fixation in valgus position to maintain good reduction with weight-bearing as done in our case have assisted in reducing avascular necrosis (AVN) risk despite the high displacement and delayed operative intervention due to the patient’s late presentation.

## Introduction

A stress fracture in general is a well-known condition that can be found commonly in healthy athletes and military personnel. It can be attributed to the training process that encompasses physical stresses and exertion [1]. A stress fracture can be classified as insufficiency or fatigue fracture on the grounds of etiology. Insufficiency fractures are commonly found in the elderly secondary to normal physiological stresses to a disease of an osseous construct such as osteoporosis and osteomalacia [2]. On the other hand, fractures occurring in normal osteology and healthy young or middle-aged individuals due to cyclic repetitive stresses can be labeled as fatigue stress fractures [3,4]. The frequency and intensity of the load exceed the bone’s ability to repair, hence the fatigue stress fracture [3]. Furthermore, the repetitive stresses will lead to skeletal damage mainly to the weight-bearing lower extremities or pelvic girdle [4]. Although stress fractures commonly occur in the tibia, they can be found in the femoral neck in some cases [5]. A well-known classification system originally published in 1961 by Garden classified neck of femur fractures depending on the anteroposterior view of the hip, dividing fracture patterns to non-displaced Garden I in which an incomplete fracture of the femoral neck with valgus impaction would be found, Garden II that is described as a non-displaced complete fracture, Garden III that is a complete fracture and partially displaced, and, lastly, Garden VI that is a complete fracture of the femoral neck with complete displacement [6]. The Garden classification was based on radiological findings; however, if we wish to classify the grade depending on the function or clinical picture of the patient, there are multiple varieties of scoring systems to assess the functional status of the hip. One of the most commonly used is the Harris Hip Score (HHS), which was applied in our case [7].

Reports on stress fractures among military personnel and active young athletes were found in previous literature [1,5,8,9]. However, this patient had an atypical history of having almost no functional limitations for seven days prior to seeking medical attention. In spite of his completely displaced neck of femur fracture, fortunately, he did not develop complications.

## Case presentation

**Figure 1:**
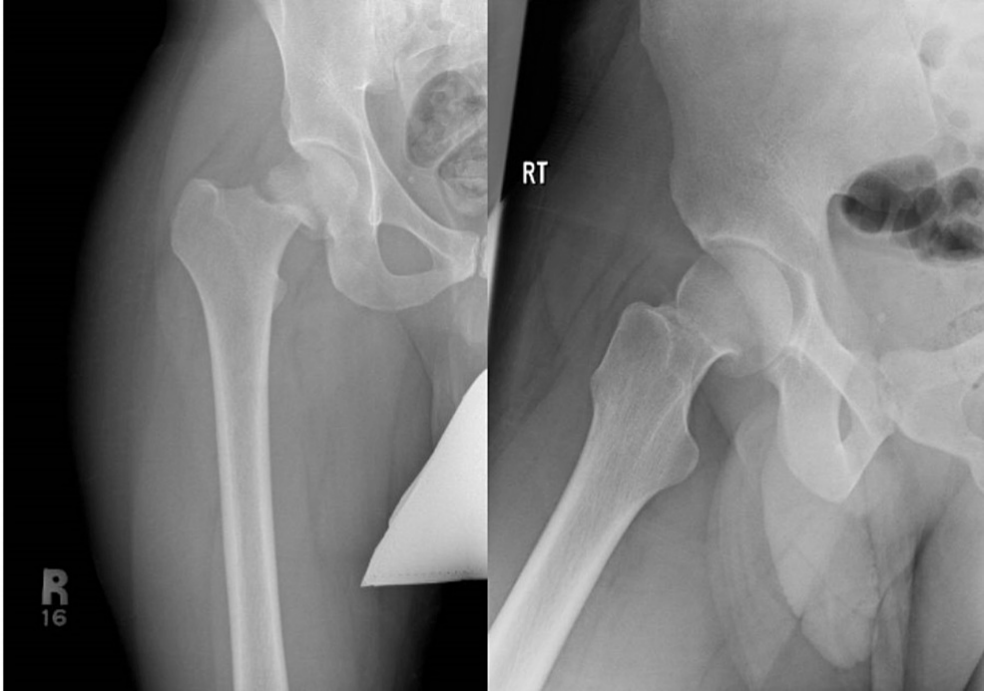
AP and lateral view X-rays of the right hip showing right displaced neck of femur fracture AP: anteroposterior

In this case, we are reporting about a 26-year-old male patient who does not have any medical illnesses, is slim, and was a very active military member during their initial period of physical training. He presented to the orthopedic surgery clinic on the 3rd of March 2018 in King Fahad Medical Military Complex complaining of right groin pain for a one-week duration that was aggravated with activity and training and relieved by rest; however, that pain did not prevent him to do his morning training or limit his usual function; he denied any history of trauma, fever, or previous episodes of the same pain. There was no limited range of motion upon examining the bilateral hips and no local signs of inflammation or swelling, and the patient can bear weight without any form of assistance. The calculated Harris Hip Score (HHS) at preoperative was 92%, putting it in the excellent group grade. Imaging in the form of X-rays was ordered and taken, revealing a right displaced neck of femur fracture (Figure 1) grade 4 when referring to Garden classification.

**Figure 2:**
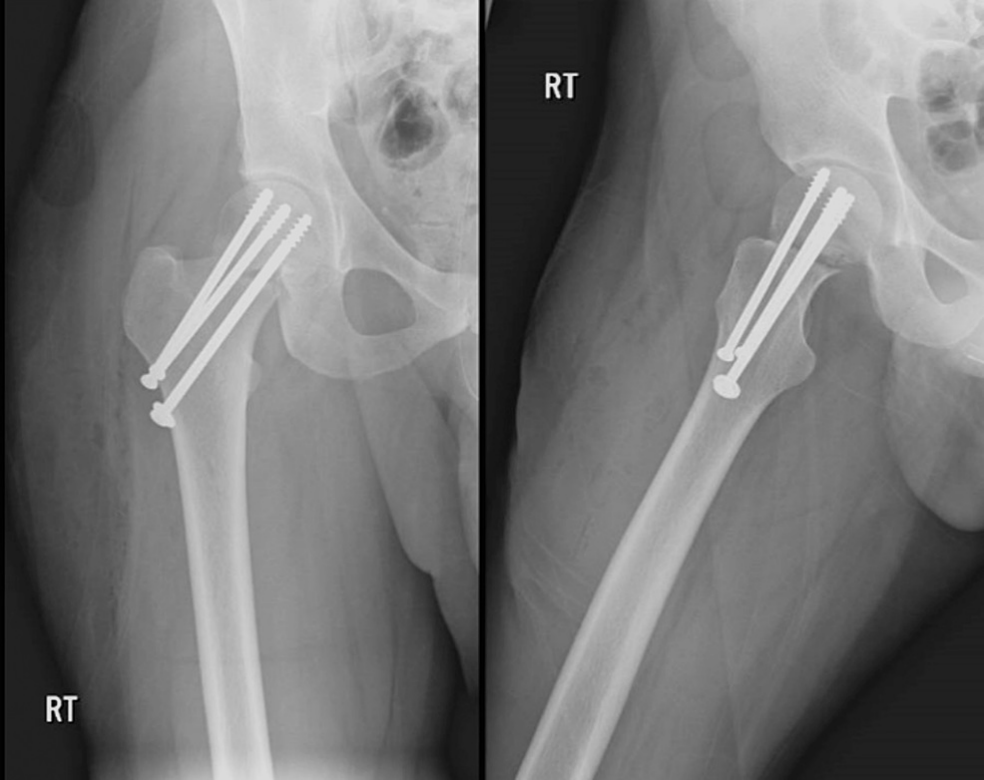
AP and lateral view X-rays of the right hip showing post close reduction and internal fixation with cannulated screws AP: anteroposterior

**Figure 3:**
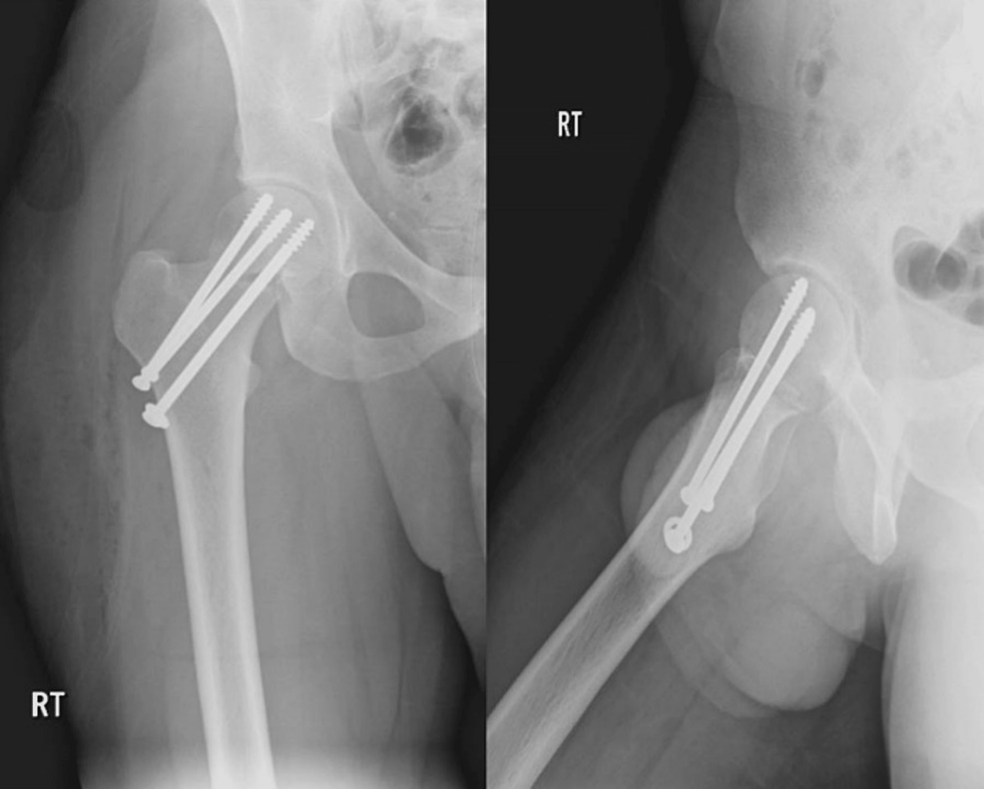
AP and lateral view follow-up X-rays of the right hip showing accepted position of screws with no displacement AP: anteroposterior

The patient was taken on the 5th of March 2018 to the operating room where he underwent close reduction in valgus position and internal fixation with three cannulated screws with a size of 7.3 in reverse triangular fashion (Figure 2). The patient was seen postoperative and was fit for discharge; in his first visit on the 22nd of March 2018, the patient had a full range of motion of the right hip, and physiotherapy with tiptoe mobilization was initiated. The calculated HHS postoperatively was approximately 77%. Anteroposterior and lateral X-rays of the hip were obtained and showed the accepted position of screws and no displacement at the fracture site (Figure 3).

**Figure 4:**
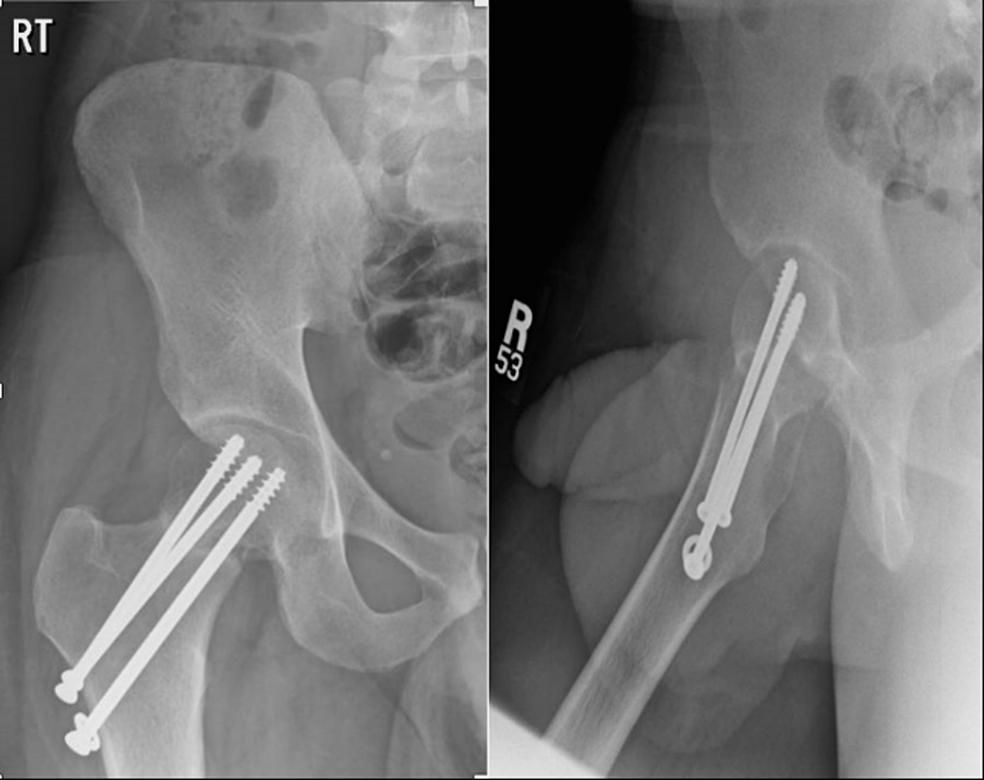
AP and lateral view follow-up X-rays of the right hip showing no signs of callus formation or union AP: anteroposterior

**Figure 5:**
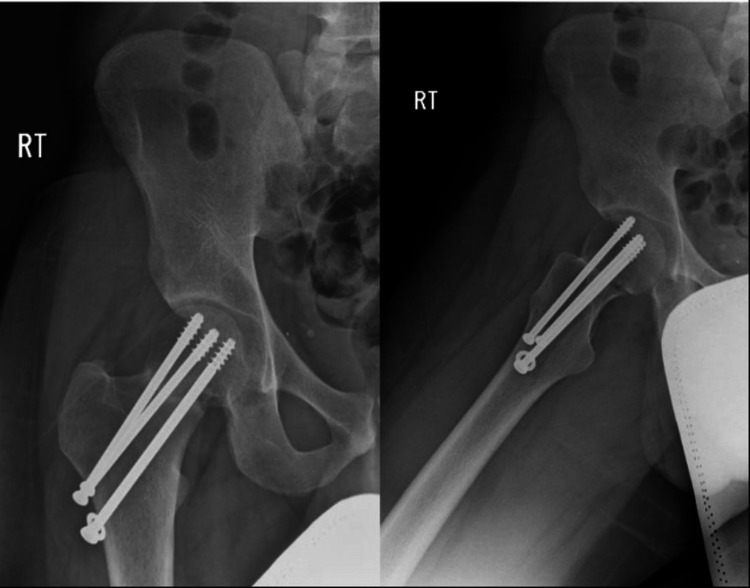
AP and lateral view X-rays of the right hip revealing signs of union in the superior aspect of the neck AP: anteroposterior

**Figure 6:**
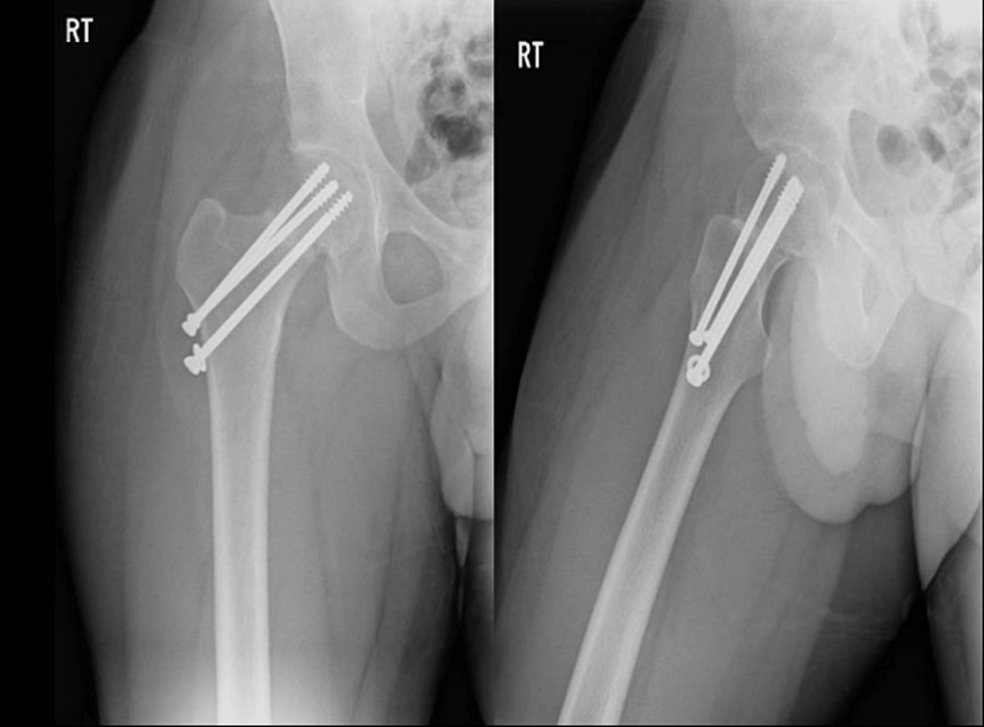
AP and lateral view X-rays of the right hip showing more signs of healing AP: anteroposterior

**Figure 7:**
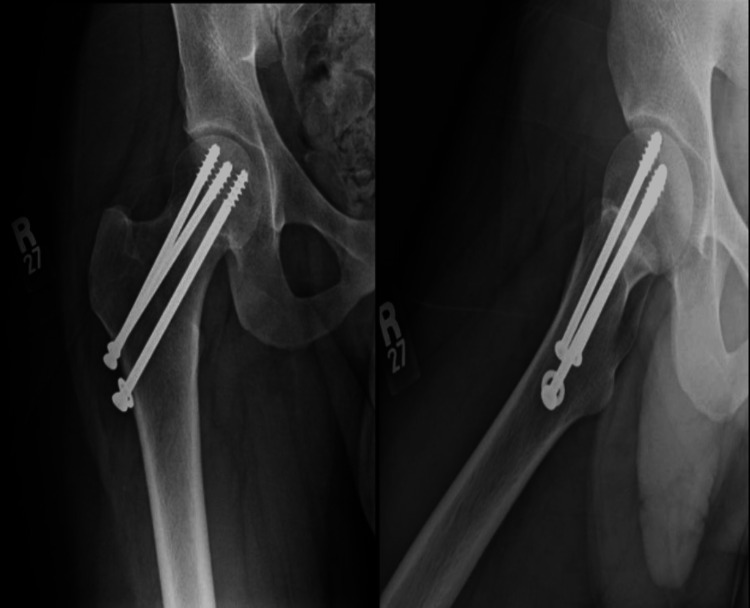
AP and lateral view X-rays of the right hip showing complete healing of the fracture with well-positioned implant with no screw cut off and no signs of head of femur avascular necrosis AP: anteroposterior

In his second visit to our clinic on the 22nd of April 2018, X-rays still showed no signs of union (Figure 4), and partial weight-bearing with crutches was initiated. In his third visit on the 17th of May 2018, X-rays revealed signs of a union in the superior aspect of the neck (Figure 5), which was followed by a later visit that showed more signs of healing as well (Figure 6). The patient was lost from follow-up until he re-presented to our clinic on the 14th of February 2019. He had returned to his usual activities and was complaining of mild pain and discomfort, with pain given 3 on a scale of 10, intermittent, and aggravated by excursion. Upon examination, he had well-healed lateral scar, no tenderness, and a full range of motion. The final calculated HHS for him was approximately 96%, which is an excellent functional result. X-rays showed complete healing of the fracture with a well-positioned implant with no screw cut off and no signs of head of femur avascular necrosis (AVN) (Figure 7).

**Figure 8:**
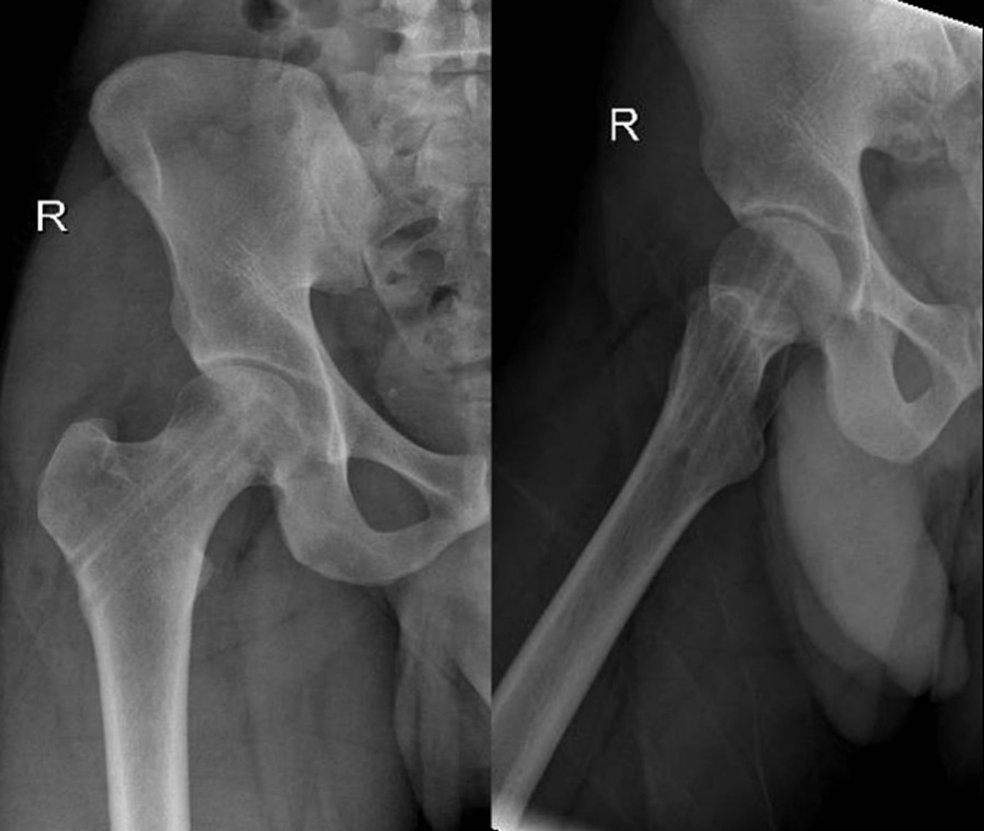
AP and lateral view X-rays of the right hip post implant removal AP: anteroposterior

**Figure 9:**
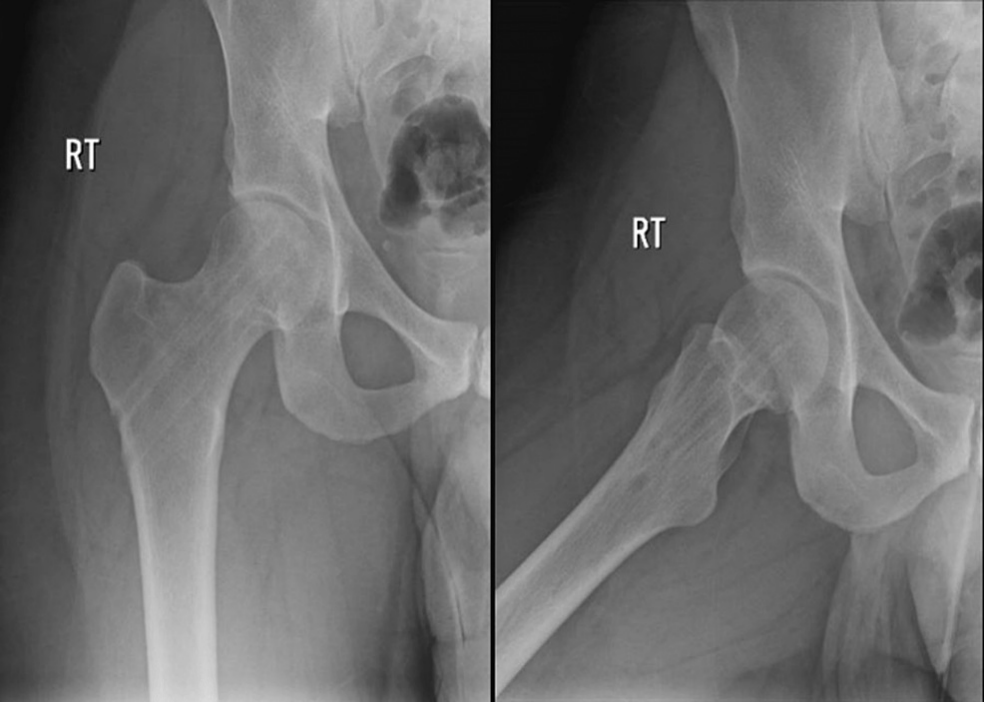
AP and lateral view follow-up X-rays of the right hip showing no signs of right head of femur AVN and acceptable bone that formed filling the screw tract AP, anteroposterior; AVN, avascular necrosis

The patient was booked for screw removal as an elective day case and underwent implant removal on the 29th of April 2021 (Figure 8). He was discharged with partial weight-bearing instructions. During follow-up after a few months after the first visit to the clinic, the images showed no signs of right head of femur AVN, and acceptable bone formed filling the screw tract (Figure 9).

## Discussion

Femoral neck stress fractures (FNSFs) occur as a result of repetitive high force or overuse injury that leads to breaking the bone partially or completely over time [1]. Such injuries can result from either abnormal stress on a normal bone (fatigue fracture) or normal stresses on the bone with deficient elasticity and structure (insufficiency fractures) [2]. These are rare injuries that account for 5% of all stress fractures. They are usually seen in osteoporosis, renal osteodystrophy, patients taking corticosteroids, and patients who engage in strenuous activities such as military recruits and marathon runners [3]. The most frequent clinical symptom is deep anterior groin pain that is exacerbated by physical exertion (running or jogging) and pain at extremes of hip movement [3]. It has been reported that clinical examination of displaced fractures results in an inability to bear weight and a shortening of an externally rotated affected limb [8]. Polacek and Småbrekke reported two cases of grade 4 displaced tension stress fracture in young active adults; on clinical examination, both of them could not bear weight [4]. In addition, a young female athlete with a displaced stress fracture was also reported. She presented initially with pain in the anterior part of the thigh, which deteriorated with training and eventually resulted in severe hip pain preventing her from walking [9]. However, in our case, the patient was completely active with no limitations of motion and able to bear weight but experienced pain with excessive activity. Therefore, he was able to train despite the fracture being completely displaced.

In addition to the clinical examination, the Harris Hip Score (HHS) was obtained at each visit. The HHS is a tool for assessing hip function across multiple domains (pain, function, the presence or absence of deformity, and the range of motion). A higher calculated score indicates less dysfunction. Each domain has a numerical score, and the total scores are calculated. The resulting range from 90 to 100 indicates an excellent result, 80 to 90 is good, 70 to 80 is fair, and less than 70 represents a poor result [7]. The clinical presentation of the patient indicates a total preoperative score of 92%, which indicates an excellent functional status with almost no deficits or dysfunction using the HHS.

In consideration of the mechanical axis of the lower extremity, the majority of the femur medial aspect would be subjected to compression, whereas the lateral aspect is under tension. As a consequence, the neck of femur enduring the repetitive load will typically result in medial aspect compression fracture or tension-type neck of femur fractures on the lateral aspect [3,6].

Femoral neck stress fractures (FNSFs) have been classified multiple times since the 1960s; the most widely used classification based on plain radiographs and bone scans is Fullerton. Fullerton classified the fracture into three categories in which type I fractures occurred on the tension side, type II fractures occurred on the compression side, and type III fractures were displacement fractures [8]. Furthermore, a fourth type was introduced by Provencher et al., a superior-based incomplete tension fracture that is commonly absent on a plain radiograph but appears on MRI [9]. A neck of femur fracture is also classified by Garden into four types ranging from incomplete fracture with no displacement to complete and fully displaced fracture based on an anteroposterior radiograph of the hip. Clinicians have simplified the classification by categorizing the fracture as non-displaced or displaced, as displacement is what most guides management plans. For displaced neck of femur fractures, fixation options include hemi/total arthroplasty or internal fixation depending on multiple factors. Grade 4 of Garden’s classification usually requires arthroplasty in the elderly, while internal fixation is still preferred in younger patients [6]. Similarly, in our case, the patient had grade 4 complete displacement in which he underwent close reduction and internal fixation with three cannulated screws with a size of 7.3 in reverse triangular fashion determined by the displacement, level of the fracture line, physiological age, pre-injury activity level, and comorbidities.

Despite the fact that most displaced fractures are not missed at presentation, a high index of suspicion is required. This is evidenced in multiple cases with a delayed diagnosis for an average of 13 weeks [10-12]. As the neck of femur is at risk for nonunion or osteonecrosis due to great strain, any displacement warrants operative management. When the femoral neck is displaced, the patient’s activity level in sports is reduced by 60%, as well as 30% at risk of developing head AVN [10]. Several studies indicate that delaying surgical decision increases the risk of AVN; as reported by Johansson et al., 30% of AVN occurred in cases of displaced FNSFs who had delayed treatment by a mean of 14 weeks versus 0% in cases who were operated on within 12 hours [10,13]. Fortunately, in our case, the patient developed neither lower levels of activities nor head of femur AVN and was spared those common complications regardless of his initial highly displaced fracture and the fact that he had it for more than seven days with no functional impact on his required vigorous training requirements as a soldier of the military.

For six weeks after surgery, the patient should be non/toe-touch weight-bearing with crutches, followed by partial weight-bearing for another six weeks; the weight-bearing is permitted as tolerated [1]. A gradual activity programmed with physiotherapist guidance is recommended, about 12 weeks postoperative focusing on improving the range of motion and strengthening around the hip joint [4,8]. Dedicated follow-up should be maintained for at least two years with clinical and subsequent radiographs in all cases to ensure that the fracture and the fixation are not losing reduction, not dislocating, or AVN [1,10]. Similarly in our case, the patient was started on tiptoe mobilization and physiotherapy from his first visit with a full range of motion followed by partial weight-bearing one month post surgery. Eventually, he returned to his normal activity level after one year of his fracture fixation.

## Conclusions

In conclusion, femoral neck stress fracture is a well-known condition that can be commonly missed in healthy military personnel with groin pain; hence, a very high index of suspicion is required especially in our case as our patient has almost no functional deficiency, and his imaging shows completely displaced fracture. The neck of femur is at risk of nonunion or AVN due to great strain, and it is an indication for operative management. Delay in the time of diagnosis and operation and the degree of displacement have been reported in the literature to increase the AVN risk. A reduction of neck of femur fracture and fixation in valgus position to maintain good and acceptable reduction with weight-bearing as done in our case has assisted in minimizing complications such as AVN risk despite the high displacement and delayed operative intervention due to the patient’s late presentation.
